# Supplementary material for: Shotgun metagenome sequencing identification of a set of genes encoded by Actinomyces associated with medication-related osteonecrosis of the jaw
Source: PLoS One. 2020 Nov 30;15(11):e0241676. doi: 10.1371/journal.pone.0241676 (PMC7703938; doi:10.1371/journal.pone.0241676)
Supplement: Attachment — Submitted filename: Response to Reviewers.docx [file pone.0241676.s004.docx]

**Point-by-point response to the editor’s and the reviewers’ comments:**

**Editor’s comments:**

Both reviewers have positive attitudes to your study. Reviewer #2, however, has pointed out that the statistical reliability of the results is minimally OK and that additional samples should be analyzed. I, not expert in the field can imagine that additional samples could be obtained and analyzed to test the frequency of the correlation.

Response: Thank you very much for your positive evaluation and constructive comments. We have substantially revised the manuscript by including additional samples as much as possible (please see our response to Reviewer #2 in detail). The extended dataset allowed us to identity two key MRONJ-associated genes that showed > 80% increase in frequency in MRONJ samples compared to their frequency in the healthy controls, and were all present only in a notable sub-clade in *Actinomyces*.

During the revision, we fixed the criterion of gene presence in the whole *Actinomyces* phylogeny to be consistent with other parts of the manuscript, and updated the figure (Figure 3, revised manuscript). We reworded “MRONJ-specific genes” as “MRONJ-associated genes” that are not completely specific to MRONJ in the extended dataset, but are still highly statistically significant.

1. Please ensure that your manuscript meets PLOS ONE's style requirements, including those for file naming.

Response: We have ensured it according to the format guidelines (<https://journals.plos.org/plosone/s/getting-started>) including the samples files to check the requirements:https://journals.plos.org/plosone/s/file?id=wjVg/PLOSOne_formatting_sample_main_body.pdf https://journals.plos.org/plosone/s/file?id=ba62/PLOSOne_formatting_sample_title_authors_affiliations.pdf

2. Thank you for including the following ethics statement on the submission details page:

'This study was approved by the ethics committees of the Research Institute National

Center for Global Health and Medicine (approval number NCGM-G-0002529-02),

National Institute of Infectious Diseases (811), Fukuoka Dental University (368).

Written informed consent was obtained.'

Please also include consent information in the ethics statement in the Methods section of your manuscript.

Response: We added “Written informed consent was obtained from the participants of the study.” to the ethics statement in the Methods section.

3. We note that you have indicated that data from this study are available upon request. PLOS only allows data to be available upon request if there are legal or ethical restrictions on sharing data publicly. For more information on unacceptable data access restrictions, please see http://journals.plos.org/plosone/s/data-availability#loc-unacceptable-data-access-restrictions.

In your revised cover letter, please address the following prompts:

a) If there are ethical or legal restrictions on sharing a de-identified data set, please explain them in detail (e.g., data contain potentially sensitive information, data are owned by a third-party organization, etc.) and who has imposed them (e.g., an ethics committee). Please also provide contact information for a data access committee, ethics committee, or other institutional body to which data requests may be sent.

b) If there are no restrictions, please upload the minimal anonymized data set necessary to replicate your study findings as either Supporting Information files or to a stable, public repository and provide us with the relevant URLs, DOIs, or accession numbers. For a list of acceptable repositories, please see http://journals.plos.org/plosone/s/data-availability#loc-recommended-repositories.

We will update your Data Availability statement on your behalf to reflect the information you provide.

Response: We made the data publicly available based on approval of the BioBank of National Center for Global Health and Medicine. We have modified the data availability statement as follows: “The sequence data after the preprocessing described below and removal of human reads of the healthy control (BioProject PRJDB10432) and those of the patients (BioProject PRJDB10606) are publicly available”.

4.PLOS requires an ORCID iD for the corresponding author in Editorial Manager on papers submitted after December 6th, 2016. Please ensure that you have an ORCID iD and that it is validated in Editorial Manager. To do this, go to ‘Update my Information’ (in the upper left-hand corner of the main menu), and click on the Fetch/Validate link next to the ORCID field. This will take you to the ORCID site and allow you to create a new iD or authenticate a pre-existing iD in Editorial Manager. Please see the following video for instructions on linking an ORCID iD to your Editorial Manager account: https://www.youtube.com/watch?v=_xcclfuvtxQ

Response: The corresponding author has linked the ORCID iD to the Editorial Manager as follows

5. Please include captions for your Supporting Information files at the end of your manuscript, and update any in-text citations to match accordingly. Please see our Supporting Information guidelines for more information: <http://journals.plos.org/plosone/s/supporting-information>.

Response: We have included the captions for Supporting Information files at the end of the manuscript, and updated any in-text citations to match accordingly.

**Response to Reviewer’s Comments**

Reviewer #1: Reviewer #1:

This paper analyzes an interesting and modern aspect of the pathogenesis of MRONJ.

The presence of actinomyces is a frequent finding in BRONJ.

Previously it was believed to be present only in osteonecrosis related to the use of bisphosphonates (BRONJ), recently it has also been detected in osteonecrosis related to denusumab (MRONJ), (as in the series that my group has just published Cerrato A, Zanette G, Boccuto M, Angelini A , Valente M, Bacci C. Actinomyces and MRONJ: A retrospective study and a literature review [published online ahead of print, 2020 Aug 20]. J Stomatol Oral Maxillofac Surg. 2020; S2468-7855 (20) 30175-0. Doi: 10.1016 / j.jormas.2020.07.012 which the Authors can quote if they want).

The present paper further supports the infectious theory of MRONJ bringing an important contribution to the knowledge of the pathology.

Response: Thank you very much for your positive evaluation and constructive comments. We quoted the interesting and timely papers (page 4, line 96).

Reviewer #2:

The manuscript by Yahara et al focusses on potential bacterial causes of MRONJ by deep shotgun metagenome sequencing of saliva samples. With the method they used, they could overcome the limitations in previous studies, such as unculturable taxa in human oral microbiome or lacking the quantitative information on abundance of specific taxa. They analyzed saliva samples collected from 5 MRONJ patients and 4 healthy volunteers, and revealed that the genus Actinomyces was 10.1% more abundant in MRONJ patients than in healthy volunteers. They also determined the 119 bacterial genes specifically found in these patients, and showed that all of them were encoded by Actinomyces genus. Furthermore they also described the most abundant eight Actinomyces genes in MRONJ patients.

In general, the data presented in this study is interesting and would shed light on an unknown field which is the molecular connection between Actinomyces and MRONJ. Nevertheless, the manuscript needs a major revision.

Response: Thank you very much for your positive evaluation and constructive comments. We have responded to your comments as follows.

Major issues

• A clear difference in bacterial composition between the samples from MRONJ patients and healthy donors could be demonstrated; however, the cohort is too small to draw any generalizable conclusions. I would suggest that at least a total of 20 samples per group should be evaluated. By taking the fact in account that MRONJ is a rare disease, the cohort can be enlarged by establishing collaborations with other centers. Still, the authors should discuss the limitations of the study, particularly because of small sample number and present their data as a pilot study in this area which needs to be confirmed further.

Response: We added salivary metagenome data of 5 other MRONJ patients we recently obtained during the peer-review of this manuscript, and those of publicly available 9 healthy controls (all the 7 samples in the Human Microbiome Project (https://www.hmpdacc.org/HMIWGS/healthy) and 2 Japanese samples). Collection of additional MRONJ samples from another hospital requires approval of its ethical committee, and the process from additional sample collection to metagenome sequencing will require more than a year. Therefore, evaluation of a total of 20 samples per group will be a subject of a future research. As suggested, we added the discussion of the limitations of the study to Abstract and Discussion sections as follows: “Although the sample size was small, this study will aid future studies to verify the data and characterize these genes in vitro and in vivo (page 3, line 51-52 in Abstract)

“Because the sample size used in this study was small, these preliminary data require further verification. (page 16, line 378-379 in Discussion)”

• Having the reads from only two MRONJ patients presented in figure S2 compromises the reliability. The authors should collect the same data from all MRONJ patients and then recreate the figure for reliable interpretation. When all these requisites have been fulfilled, Figure S2 may be one of the main figures since determination of Actinomyces-specific genes found predominantly in MRONJ patients is an important research question in this study.

Response: As suggested, we collected the same data from all the ten MRONJ patients and then recreated it as a main figure (Figure 2A, revised manuscript, shown below) for 31 genes that were found to be significantly associated with MRONJ in the extended dataset. When the 31 genes were ranked based on the sum of abundance (TPM values that could be directly compared among the samples because the sum of the values was normalized to 1 million) om the 10 patients, the top two genes were *gcpE* and *dps* that were found to be all present only in a sub-clade of *Actinomyces* (Figure 3 and Figure 4, revised manuscript). We really appreciate your insightful comment that enabled the discovery.

• The manuscript is well-written and easy to follow except from the discussion part. This section should be more compactly structured to lead the reader to follow the story depicted in Figure 4.

Response: As suggested, we have made the discussion part compactly structured by focusing on the top two genes.

Minor issues

• It would be easier to follow if the figure legends were presented with the figures in the manuscript.

Response: According to the guideline of PLOS ONE (as shown below), the figure legends are placed “directly after the paragraph in which they are first cited”

• There’re typos in Figure S2 axis labeling.

Response: We have fixed the typos to be “patient”.

• Lines 278-280: …orange and purple indicate presence and absence of the genes. Missing “, respectively”?

• Line 285: “except” instead of “outside”

Response: We have added them accordingly.
